# The Aha! Glow: Insight Experiences Bias Judgements of Idea Quality in Divergent Contexts

**DOI:** 10.3390/jintelligence14080160

**Published:** 2026-08-01

**Authors:** Jacqueline E. McGuinness, Jonathan W. Schooler, Shelly L. Gable, Madeleine E. Gross

**Affiliations:** 1 Department of Psychological and Brain Sciences, University of California Santa Barbara, Santa Barbara, CA 93106, USA; jemcguinness@ucsb.edu (J.E.M.); jschooler@ucsb.edu (J.W.S.); sgable@ucsb.edu (S.L.G.); 2 Department of Psychology, Loyola Marymount University, Los Angeles, CA 90045, USA

**Keywords:** insight, creativity, divergent thinking, convergent thinking, individual differences

## Abstract

One of the defining characteristics of the Aha! experience is the profound sense of certainty with which it arrives—you don’t just know, you *know you know*. A large body of research suggests this confidence is generally warranted; ideas that arrive with a feeling of insight are more likely to be accurate than those that do not. However, the insight–accuracy relationship has been examined almost exclusively in convergent thinking tasks, leaving the diagnostic value of insights in divergent contexts uncertain. The present research tests whether this relationship differs between problem-solving contexts. Study 1 re-analyses daily diary data from professional writers and physicists, used as proxies for divergent and convergent domains. Writers initially rated their Aha! ideas as highly creative but downgraded them at 6-month follow-up; physicists’ Aha! ideas, by contrast, retained or gained value over time. Study 2 used a within-subjects design with laboratory measures of convergent and divergent thinking. Insight intensity predicted accuracy on both tasks but predicted subjective creativity ratings far more strongly than objective ones on the divergent task, producing a systematic bias we term the *Aha! glow*. Implications are discussed, including a proposed adaptive, motivational function for this phenomenon.

## 1. Introduction

One of the most coveted experiences in the creative process is the “Aha!” (Lucas & Nordgren, 2022), that satisfying moment when an idea suddenly clicks into place, engendering a sense of excitement and drive. Perhaps the most unique feature of the insight experience is the profound sense of certainty it arrives with: you don’t just know, you *know you know.* But is the Aha! just a pleasant afterglow of having solved a problem, or does it genuinely signal the quality of the ideas it accompanies? In this research, we examine how reliably insights signal idea quality across creative contexts.

A large and growing body of work suggests that insight experiences may hold genuine diagnostic value; solutions accompanied by a feeling of insight are more likely to be correct compared to those reached through more strategic or analytic processes. This pattern is remarkably consistent across different problem types, including classic insight problems, magic tricks, anagram tasks, rebus puzzles, ambiguous visual images, and compound remote associates (CRAs) (Danek & Wiley, 2017; Hedne et al., 2016; Salvi et al., 2016; Webb et al., 2016).

The convergence of these findings led to the development of the Eureka Heuristic, a theoretical framework proposing that insights function as metacognitive shortcuts that help individuals to rapidly evaluate the quality of newly developed ideas (Laukkonen et al., 2023). During problem-solving, unconscious processes work in the background to integrate information and test possible solutions against accumulated knowledge. An Aha! moment, according to this framework, occurs when these processes culminate in a potential solution; the phenomenological experience of the Aha!, in turn, functions to capture attention and encourage quick action, sidestepping the need for slower, more deliberative thinking. The central premise of the Eureka Heuristic is that when our implicit knowledge is sound, insights should reliably signal the accuracy of our ideas. Conversely, if information is corrupted (e.g., misinformation, false memory, semantic priming, etc.), reliance on the insight signal could result in poor or incorrect ideas.

Indeed, past research has shown that in certain circumstances, the feeling of insight can mislead judgments of idea accuracy. For example, the phenomenology of insights can be misattributed to temporally coincident but unrelated information—a phenomenon referred to as “Aha! misattribution.” One paradigm asked participants to evaluate true/false claims in which a key word was either presented as a scrambled anagram (e.g., “iliuthm is the lightest of metals”) or presented straightforwardly (“Lithium is the lightest of metals”; Laukkonen et al., 2020). After either solving the anagram or being given the solution, participants rated how likely it was that the statement was true. Successfully solved statements, compared to non-anagram statements, received the highest truth ratings, regardless of whether the statements were true or false. Critically, these effects were strongest when solving the anagram engendered an Aha! experience. In other words, the Aha! experience from solving the anagram was misattributed as a signal of truthiness when temporally linked with unrelated information, causing people to endorse ideas that do not merit such confidence.

More recently, researchers have begun to theorize that insights under the influence of psychedelics might similarly be vulnerable to heightened misattribution (McGovern et al., 2024). For instance, one theory suggests that psychedelic compounds (e.g., psilocybin and LSD) relax prior beliefs and therefore reduce one’s ability to make sense of incoming sensory information. This results in a high number of insights and strong noetic experiences, which crucially, are not selected for due to the accuracy or adaptiveness of such ideas (McGovern et al., 2024). Furthermore, evidence suggests that psychedelic use has been linked to major life changes following a sudden change in beliefs—including changes to relationship status, diet or exercise, religious or spiritual beliefs, and career status—the health benefits of which vary depending on the context and individual (Carlisle et al., 2026). Thus, in addition to the influence of misleading contextual information, altered mental states induce particularly strong insight phenomenology, which can be misattributed to ideas as a signal of veracity.

A daily diary study offers suggestive evidence that insights can also bias perceptions of idea quality. Gable et al. (2019) explored how insights predict idea quality across two occupational domains: creative writing and physics. Each day for 14 days, 53 professional writers and 45 professional physicists were asked to report their most creative idea, whether the idea was accompanied by an Aha!, and how creative they thought the idea was. At 6 month follow-up, participants were asked to rate how creative those same ideas ended up being. Ideas accompanied by an Aha! were rated as significantly more creative than non-Aha ideas at the time of idea generation; at 6-month follow-up, however, Aha! ideas showed a significantly greater decline in creative value. In other words, Aha! ideas were less able to “stand the test of time,” such that perceptions of their creative value decreased over 6 months. This pattern provides initial evidence for the existence of what we term an *Aha! glow*, which refers to instances in which the phenomenological experience of insight leads people to overestimate the creative value of their ideas at the time of generation.

Although past research suggests that the diagnostic value of insights is at the very least fallible, this may not be reflected in people’s lay beliefs about the value of insight solutions. Across several studies, individuals have been found to reliably overestimate the role insights play in contributing to creative outputs as compared with analytical reasoning (Lucas & Nordgren, 2022). Put another way, individuals tend to think that most creative work stems from moments of insight, rather than from effortful, sustained engagement with creative work—a lay belief Lucas and Nordgren term “insight bias.” In short, not only does past research suggest that, in certain contexts, the phenomenological experience of insight can be misattributed as a signal of accuracy, but also that individuals tend to believe insights play a larger role in creative output than they actually do.

Creativity research distinguishes between convergent thinking, in which individuals are challenged to arrive at a single, correct solution to a problem, and divergent thinking, in which individuals generate multiple ideas that vary in their creative merit. This distinction may be consequential for whether insights genuinely act as a signal of idea quality. In convergent contexts, where there is a genuine answer, it may be possible for individuals to compare their generated solutions against an implicit marker or existing latent knowledge, as proposed in the Eureka Heuristic framework. In divergent contexts, where the creative value of ideas often emerges over time as the idea receives public recognition, our immediate, subjective perceptions of idea quality might be more vulnerable to bias. In other words, we might expect creative insights during divergent problem-solving to be more vulnerable to the *Aha! glow*, or other contextual factors that have been shown to bias the diagnostic value of insight in convergent contexts (Laukkonen et al., 2020).

Research has only recently begun to explore insight phenomenology within divergent thinking problems (Smith et al., 2026). One such study examined the rate of reported insight experiences within category generation tasks and alternate uses tasks, which are well-established measures of divergent thinking. They found that Aha! experiences occurred in approximately one in four responses on the alternate uses tasks. Furthermore, Aha! ideas were rated as more creative and novel compared to non-Aha ideas and were also associated with longer time lapses between successive responses—referred to as the “nascent period” which directly precedes an insight idea (Smith et al., 2026). Taken together, these findings were the first to show that insight experiences reliably occur in divergent thinking contexts and suggest that insights may also be indicative of novel solutions on such problems.

Another study explored how individual differences in divergent thinking ability relate to insight experiences and perceived idea quality. They found that higher originality scores on a divergent thinking measure predicted greater trial-level insight phenomenology across a range of classic insight, non-insight, and compound remote associate problems. However, originality scores did not predict solution accuracy within any of these problem types (Webb et al., 2021). In other words, for creative individuals (at least of divergent type creativity) the established relationship between insight intensity and solution accuracy on convergent creativity problems may be less straightforward. Crucially, to our knowledge, no study has directly explored whether the relationship between insight intensity and idea quality differs depending on whether the problem-solving context itself is divergent or convergent.

Furthermore, while the diagnostic value of insights in divergent contexts remains an open question, past research suggests they may play another important role, namely, to motivate individuals toward further discovery and exploration. Aha! experiences are associated with epistemic feelings and emotions, such as ease, confidence, suddenness, and positive affect, which, in turn, can foster feelings of motivation and renewed self-efficacy within a domain of interest (for review, see Prenevost & Reber, 2024). In the context of divergent thinking, this role may be especially valuable given that there are often few external sources of feedback or validation until the idea is distributed for public evaluation. It is therefore plausible that insights might serve a greater motivational function in divergent contexts, helping to sustain engagement, refinement, and development of creative ideas, even when there is no immediate guarantee of success.

### Study 1

The initial findings reported in Gable et al. (2019) showed that ideas that were classified as Aha!’s saw a systematic decline in perceived creativity over time, suggesting the presence of an *Aha! glow*: the Aha! experience may cause individuals to overestimate the quality of their idea at the time of generation. Crucially, the original study did not examine whether this phenomenon differed between professions. Professional physicists tend to work on problems that have a single, defined solution, which can be verified based on prior knowledge or formal analysis. Indeed, Kuhn (1962) famously argues that “normal science” is like solving a jigsaw puzzle or crossword—each problem has a guaranteed solution, such that the challenge lies within the skill of the scientist to find an answer within a given paradigm. This line of reasoning then suggests that physicists tend to work on convergent creativity problems. On the other hand, professional writers face creative problems in which many possible ideas can lead to a viable product—akin to divergent thinking problems, which are ill-defined and have many acceptable solutions (Guilford, 1967b). Thus, taken together, physics and creative writing appear to reside on opposite ends of a problem-solving continuum from convergent to divergent thinking domains, respectively.

The Gable et al. (2019) sample therefore provides an ecologically valid opportunity to compare the diagnostic value of insights within real-world convergent and divergent problem-solving contexts. In the Supplementary Materials of the original study, they report that physicists showed a significant increase in the creativity ratings of their ideas overall, while writers showed a non-significant decrease over time. This provides suggestive evidence that individuals who work on divergent thinking problems experience an initial bias in their perceptions of idea quality, which declines over time.

Based upon these initial findings, our key novel prediction was that the previously observed decline in creativity ratings over time would be specific to the Aha! ideas of writers. Specifically, we predicted a significant three-way interaction between profession, idea type, and time, such that the Aha! ideas of writers would show a significant decline in self-reported creativity over time, while the Aha! ideas of physicists would remain constant. Crucially, we predicted that this pattern would not be observed for non-Aha ideas; the ratings for non-Aha ideas were predicted to remain stable for both writers and physicists over time. Confirmation of these predictions would provide the first evidence that the reliability of insights as a signal of accuracy depends on the problem-solving context, placing an important caveat on the Eureka Heuristic framework of insight.

Gable et al. (2019) also collected several daily measures of motivation, which were not explored in the original study, including measures of inspiration, self-confidence, and time spent working on creative pursuits. Therefore, to explore the potential motivational role of insights, and whether this function is particularly strong in divergent thinking contexts, we also tested whether days in which people experience an insight are associated with greater motivational outcomes. Furthermore, we explored whether the strength of this relationship differed between professional writers and physicists.

## 2. Materials and Methods

### 2.1. Dataset Overview

This study is a secondary analysis of the existing dataset from Gable et al. (2019), which probed a sample of professional writers and physicists about their topmost creative ideas each day. Our central research question is whether the relationship between insight and accuracy differs between physicists and writers, who are taken to represent convergent and divergent problem-solving contexts. This reflects a distinction from the original study, where writers and physicists were grouped together for the main analyses.

The original research consisted of two studies, both of which used a daily diary study design with a follow-up questionnaire. The present research reanalyzed the data from study 1 of Gable et al. (2019), which sampled an approximately equal number of writers and physicists. The sample from study 2, by contrast, was substantially overrepresented by writers (*n* = 60) compared to physicists (*n* = 27). This raised concerns about imbalanced precision and unstable estimates for the physicists in our analyses of group comparisons, as the proportion of writers in the sample is nearly double that of physicists. Furthermore, there were key methodological differences between the two studies. In study 1 of Gable et al. (2019), daily diary data was collected over 2 weeks, and participants were probed again 6 months later. On the other hand, study 2 collected daily diary ideas over only one week, and participants were probed again at 3 months follow-up. Our primary goal in the present research is to compare the association between insight intensity and idea quality across professions under roughly similar conditions of statistical power. Furthermore, our measure of idea quality is the pattern of change in self-reported creativity ratings over time, which requires substantial length between the time of idea generation and follow-up questionnaire. Thus, for the analytic and design considerations mentioned above, we restricted our analysis to study 1.

### 2.2. Participants

Participants in study 1 of Gable et al. (2019) were composed of 45 physicists and 53 professional writers (*N* = 98). The sample of physicists was recruited from the Kavli Institute for Theoretical Physics at the University of California Santa Barbara, and consisted of 8 females and 37 males, with a mean age of 35.07 years. Professional writers were recruited through advertisements in the Writers Guild of America’s quarterly magazine. To meet the criteria for inclusion in the study, participants were required to report spending at least 20 hours per week on writing. The sample of writers consisted of 28 females and 25 males, with a mean age of 49.65 years. All participants were paid $150 for completing the daily diary study, and an additional $50 for the follow-up survey.

### 2.3. Measures

#### 2.3.1. Idea Quality (Time 1)

Every evening, participants were asked to rate, “How creative do you think this idea is?” on a scale from 1 (*slightly creative*) to 7 (*extremely creative*).

#### 2.3.2. Aha Experience

After reporting their most creative idea, participants were asked to indicate “Yes” or “No,” whether the idea felt like an Aha! moment.

#### 2.3.3. Idea Quality at Follow-Up (Time 2)

At 6-month follow-up, participants were shown all of their responses describing their top most creative ideas from the 2-week daily diary portion. For each response, they were asked to rate again, “How creative do you feel the idea was?” on a scale from 1 (*slightly creative*) to 7 (*extremely creative*).

#### 2.3.4. Daily Motivational Outcomes

In addition to rating idea quality, every night of the two-week daily diary portion, participants were also asked to rate their levels of inspiration, self-confidence, and time working on creative projects through the course of the day. Participants were asked to self-report their feeling of inspiration on a scale from 1 (*not at all inspired*) to 5 (*extremely inspired*). To assess self-confidence, participants were asked to rate their agreement with the statement, “Today, I felt more self-confident than usual” on a scale from 1 (*strongly disagree*) to 5 (*strongly agree*). Finally, participants were asked to respond, “How much time did you spend specifically working on problems pertaining to your personal interests,” on a scale from 1 (*none at all—0 hours*) to 11 (*9 or more hours*).

### 2.4. Procedure

The study procedures are outlined in Gable et al. (2019) and are summarized below. After providing informed consent and demographic information, participants were emailed a survey every night for two weeks. At the end of each day, participants were asked to report the most significant, creative idea they had, but were reminded that creative ideas are not expected to be generated every day, and given the option to skip this part of the survey. If they responded with an idea, participants were then asked to answer several questions about the idea generation, along with questions pertaining to their mood and behavior throughout the day. Importantly, the responses were coded by two trained researchers to verify whether they constituted a valid creative idea (e.g., responses like “I was too busy today” were excluded).

After 6-months, participants were emailed again to complete the follow-up survey, in which they were shown all the creative ideas they listed during the daily diary portion of the study. They were asked to rate each idea retrospectively in terms of creativity and importance. Participants were sent up to 5 reminders to complete the follow-up survey, and the rate of completion for the follow-up questionnaire was 94%.

## 3. Results

### 3.1. Analytic Strategy

Because the daily diary data have a hierarchical structure, with multiple ideas (Level 1) nested within participants (Level 2), we used linear mixed modeling methods to analyze the data, in line with the approach used in Gable et al. (2019). All models were estimated using SPSS (version 30.0).

Our primary objective was to analyze the change in creativity ratings over time by profession and type of idea. To examine this pattern, a linear mixed effects model was fit with profession (writer vs. physicist), type of idea (Aha! vs. non-Aha), and time (T1 vs. T2) entered as fixed effects, and participant ID entered as a random effect (intercepts only). We then examined the three-way interaction between our three fixed effects and the outcome variable: creativity scores. We decomposed the three-way interaction with simple effects, which were derived from the same model via custom contrasts.

To replicate the findings from Gable et al. (2019), we also explored all main effects and two-way interactions, which are reported in Supplementary Materials. Because this model estimates all main effects, two-way interactions, and the three-way interaction simultaneously, the lower-order effects reported in the present study are adjusted for the higher order terms. Thus, the numeric values of our effects differed slightly from the original published results. However, we note that the pattern of findings from all main effects and two-way interactions within the three-way interaction model replicated the results from the original study (see Supplementary Materials).

### 3.2. Is the Decline in Aha! Ratings Specific to Writers?

In line with our key hypothesis, the model revealed a significant Profession × Idea Type × Time interaction, *F*(1, 1349.21) = 5.53, *p* = .019, see Table 1 for cell means. We decomposed this interaction in two complementary ways.

#### 3.2.1. Simple Idea Type × Time Interactions Within Each Profession

Within writers, the Idea Type × Time interaction was significant, *b* = −0.86, *SE* = 0.18, *t*(1342.54) = −4.69, *p* < .001: Aha! ideas dropped in creativity ratings from T1 (*M* = 5.07) to T2 (*M* = 4.59), *b* = −0.38, *SE* = 0.13, *t*(1343.00) = −2.94, *p* = .003, while non-Aha ideas rose in the opposite direction (T1 *M* = 3.95; T2 *M* = 4.33), *b* = 0.48, *SE* = 0.13, *t*(1346.95) = 3.69, *p* < .001. Within physicists, the Idea Type × Time interaction was non-significant, *b* = −0.17, *SE* = 0.23, *t*(1353.29) = −0.72, *p* = .47: Aha! ideas rose in ratings from T1 (*M* = 3.98) to T2 (*M* = 4.45), *b* = 0.48, *SE* = 0.19, *t*(1356.28) = 2.55, *p* = .011, and non-Aha ideas also rose (T1 *M* = 3.16; T2 *M* = 3.80), *b* = 0.64, *SE* = 0.14, *t*(1352.27) = 4.67, *p* < .001.

#### 3.2.2. Simple Profession × Time Interactions Within Each Idea Type

For Aha! ideas, the Profession × Time interaction was significant, *b* = −0.96, *SE* = 0.23, *t*(1353.26) = −4.20, *p* < .001: writers’ ratings fell from T1 (*M* = 5.07) to T2 (*M* = 4.59), *b* = −0.38, *SE* = 0.13, *t*(1343.00) = −2.94, *p* = .003, whereas physicists’ ratings rose from T1 (*M* = 3.98) to T2 (*M* = 4.45), *b* = 0.48, *SE* = 0.19, *t*(1356.28) = 2.55, *p* = .011—a clear crossover. For non-Aha ideas, the Profession × Time interaction was non-significant, *b* = −0.26, *SE* = 0.19, *t*(1347.97) = −1.39, *p* = .17: both groups showed increases over time (writers: +0.48, *b* = 0.48, *SE* = 0.13, *t*(1346.95) = 3.69, *p* < .001; physicists: +0.64, *b* = 0.64, *SE* = 0.14, *t*(1352.27) = 4.67, *p* < .001), and these increases did not differ reliably between professions.

**Figure 1:**
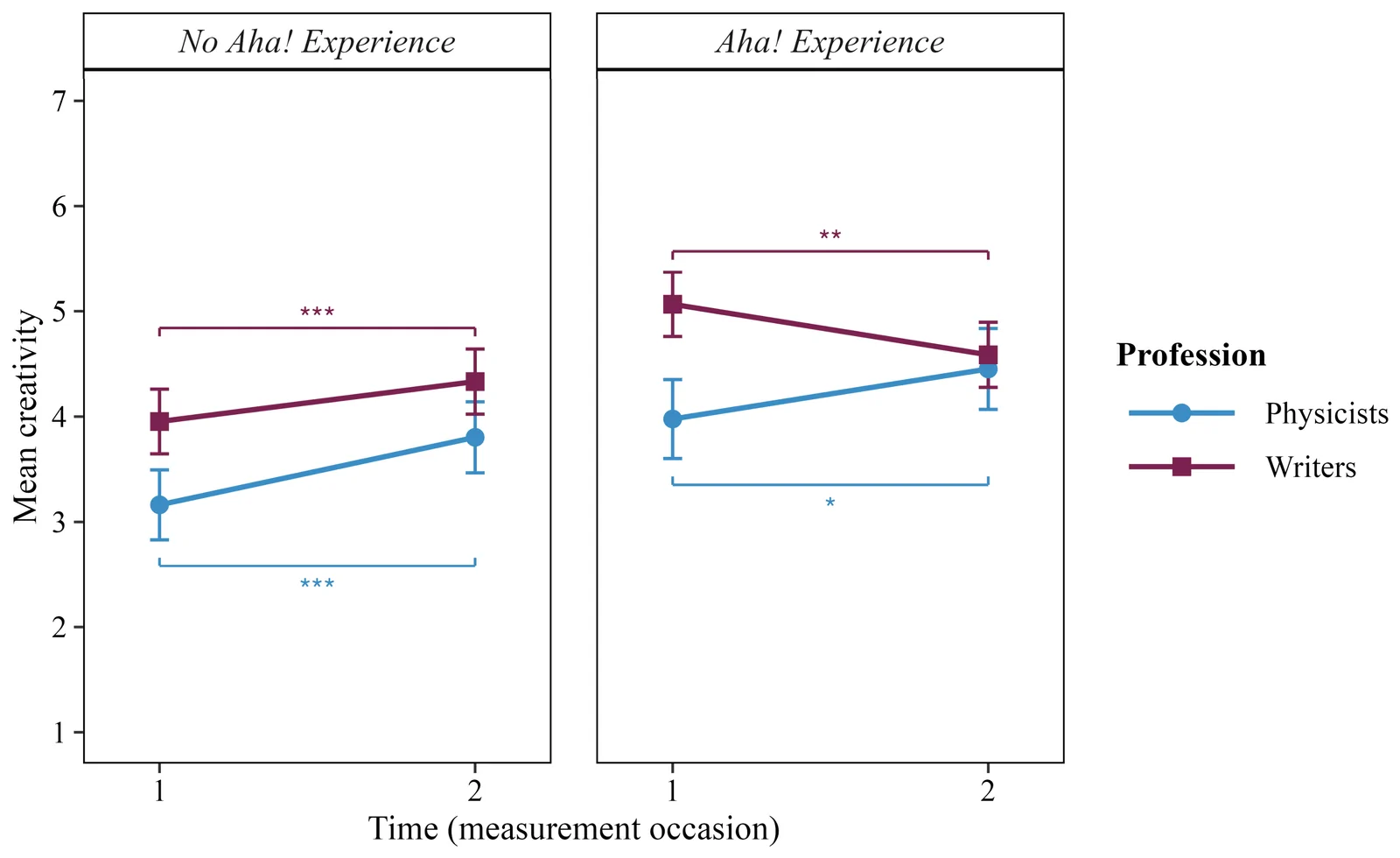
Changes in Creativity Over Time by Profession and Idea Type. Note. Estimated marginal mean creativity scores at Time 1 and Time 2 for physicists and writers, separated by Aha! experience. Error bars represent 95% confidence intervals. * *p* < .05, ** *p* < .01, *** *p* < .001.

In summary, the results from Study 1 extend the findings of Gable et al. (2019), and reveal a significant crossover interaction that is specific to Aha! ideas (see Figure 1).Writers initially overestimate the creativity of their Aha! ideas but show a decline in ratings at 6-month follow-up. Physicists, on the other hand, initially rate their Aha! ideas as less creative than writers but come to value them more over time. Importantly, this pattern is not observed for non-Aha ideas—both writers and physicists rate their analytic ideas slightly more strongly over time and to a similar degree. This novel finding suggests that the feeling of Aha! inflates perceived creativity for people working in divergent problem-solving contexts (like writers), but not for people working in convergent problem-solving contexts (like physicists).

### 3.3. Motivational Correlates of the Aha! Experience

In addition to our central research question, we also conducted supplementary analyses to explore whether insight experiences on a given day predict greater inspiration, self-efficacy, and time spent working on creative projects. Furthermore, we tested whether the association between insight and motivation differs by profession. To explore this question, we fit several linear mixed effect models with idea type (Aha! vs. non-Aha) and profession (physicist vs. writer) as fixed effects, and participant ID as a random effect. Three separate models were run with each of the three motivational outcomes as dependent variables. The interaction between idea type and profession was tested to explore whether the association between insight and motivation depends on idea context.

First, we explored whether participants experience greater motivation, as captured by nightly reports of inspiration, self-confidence, and time spent working on creative projects, on days in which they reported an Aha! experience. A linear mixed effect model revealed that participants reported significantly higher inspiration on days in which they experienced an Aha! moment (*M* = 3.34), compared to days in which they had no Aha! (*M* = 2.95), *b* = −0.34, *SE* = 0.086, *t*(732.77) = −3.96, *p* < .001. However, the model did not reveal a significant interaction between idea type and profession on self-reported inspiration, *b* = −0.096, *SE* = 0.14, *t*(737) = −0.69, *p* = 0.49. So, we do not find evidence that the association between creative insights and inspiration is stronger for writers compared to physicists.

Similarly, a second linear mixed effect model revealed a significant main effect of idea type on self-confidence, *b* = −0.28, *SE* = 0.079, *t*(741) = −3.51, *p* < .001. On days in which participants reported an Aha! experience, they also reported higher levels of self-confidence (*M* = 3.26) compared to days without an insight experience (*M* = 3.06). However, there was again no significant interaction between idea type and profession on self-confidence, *b* = 0.15, *SE* = 0.13, *t*(741) = 1.15, *p* = 0.25.

Finally, a linear mixed effects model revealed that people reported a greater amount of time spent working on relevant creative projects on days when they had an Aha! idea (*M* = 4.73), compared to days with no Aha! (*M* = 5.28), *b* = −0.39, *SE* = 0.20, *t*(711.49) = −2.01, *p* = 0.045. Once again, there was no significant interaction between idea type and profession on time spent working on creative projects, *b* = −0.32, *SE* = 0.32, *t*(723.91) = −1.01, *p* = 0.31.

In sum, results show that on days in which participants reported an Aha! experience, they tended to report higher levels of inspiration, self-confidence, and time spent working on creative projects. However, this effect does not appear to be stronger in divergent thinking contexts (i.e., writers), compared to convergent thinking contexts (i.e., physicists). In other words, we do not find evidence that the motivational role of insight is elevated in divergent thinking contexts.

## 4. Interim Discussion

Using the rich set of daily diary data obtained by Gable et al. (2019), Study 1 tested whether the relationship between insight and idea quality differs depending on the problem-solving context. Type of occupational profession was used as a proxy for divergent and convergent problem solving, allowing us to explore whether the previously reported decline in the perceived creative merit of ideas associated with insight generalizes across creative contexts.

The results demonstrated that the previously observed decline in creativity ratings for Aha! ideas was, in fact, specific to writers. Professional writers initially rated their Aha! ideas as highly creative, but their ratings significantly declined over the 6-month period; the ratings of professional physicists did not demonstrate this pattern. Indeed, physicists came to view their Aha! ideas more favorably over time. Importantly, this crossover pattern only held for ideas accompanied by a feeling of Aha!; non-Aha ideas increased in creative value over time, and this pattern did not meaningfully differ between occupational groups. If professional writers and physicists are viewed as real-world proxies for divergent and convergent problem-solving contexts, this crossover interaction provides initial evidence that the Aha! experience might not uniformly serve as a signal of idea quality across problem types. Specifically, results suggest the existence of a heightened *Aha! glow* among divergent thinking contexts, in which the feeling of insight inflates perceptions of idea quality.

The present findings add an important dimension to the Eureka Heuristic framework, which posits that the insight experience works by helping individuals to quickly discern the veracity of new ideas (Laukkonen et al., 2023). This framework is consistent with the pattern demonstrated by the physicists in the sample, where the merit of their ideas held up, or even gained value, over time. Physicists typically work on convergent creativity problems, where there is one correct solution which can be evaluated based upon prior knowledge or other aids (e.g., formal analysis). On the other hand, in divergent contexts, where there is no objective benchmark to compare ideas to at the time of idea generation, insights may be less reliable. Consistent with this, writers were found to rate their ideas as more creative at the time of generation.

One plausible interpretation of these results is that, in divergent contexts, the insight signal may be more susceptible to the vulnerabilities observed in the previously described “Aha! misattribution” paradigm (Laukkonen et al., 2020). Without an objective benchmark to evaluate ideas against, the affective punch of the insight experience fosters an inflated perception of idea quality—or *Aha! glow*, as we refer to in the present research—even in cases where the idea does not genuinely warrant such confidence. In other words, the perceived quality of the idea may be an artifact of the phenomenology of the insight itself. Of course, Aha! ideas still hold value in divergent contexts; rather, the present findings suggest that the magnitude of the perceived creativity may be both overestimated and partly attributable to the phenomenological experience of the insight itself. Consistent with this, at 6-month follow up, writers rated their Aha! ideas marginally more creative than non-Aha ideas, indicating a weak, but potentially still present diagnostic value of Aha! experiences in divergent contexts.

While the phenomenological impact of insights may render them less reliable in divergent contexts, they may still serve a valuable motivational role. The results of Study 1 showed that on days when participants had an Aha! experience, they reported greater inspiration, self-confidence, and time spent working on relevant creative projects, compared to days in which they had no Aha! experience. However, we did not find that this effect was especially pronounced for writers (i.e., roughly, divergent thinking contexts). Importantly, these effects were correlational, so it may also be the case that people were more likely to have an insight when they felt more motivated, or that some third variable (e.g., mood) was responsible for both insight and motivation. Nonetheless, the present research provides suggestive evidence that the Aha! experience serves two important functions in problem solving: (1) an epistemic role, signaling individuals toward accurate solutions and (2) a motivational role, sustaining individuals’ pursuit and refinement of potentially creative ideas. Taken together, these results suggests that, for writers, insights may inflate assessments of creative merit but, in so doing, provide an adaptive motivational role. Specifically, insights may provide the necessary encouragement to continue pursuing ideas that cannot yet be externally validated. This function of insights may be particularly beneficial in divergent fields where there is no clear compass for gauging progress, yet where sustained engagement may eventually lead to genuinely creative outputs.

There are several important limitations of Study 1. Perhaps the most critical for the present research focus is that professional writers and physicists were used as imperfect proxies for divergent and convergent creativity domains. The strength of preserving ecological validity bears the cost that, on any given day, physicists might engage with a divergent creativity problem (e.g., the formulation of a new theory); or conversely, writers might encounter a convergent creativity problem. Furthermore, profession is a between-subjects variable that can be confounded by other factors, such as workplace environment, personality, expertise, or other demographics (e.g., education level). More specifically, any other differences between professional physicists and writers, beyond the convergent-divergent distinction, may hypothetically contribute to the observed patterns. Relatedly, it is possible that the effects of Study 1 were driven by individual differences in creative thinking style, rather than idea context. Put simply, it is possible that the insight–accuracy association depends on whether people are convergent or divergent *thinkers*, rather than the specific problem-solving context. To address these central limitations, we ran an additional study to compare the insight–accuracy relationship directly across divergent and convergent problem-solving contexts.

One final limitation of Study 1 is that the sample of professional physicists is majorly comprised of men—of the 45 physicists from whom data was collected, only eight participants were female. Although this gender discrepancy might reflect a meaningful difference in the rate of men versus women who enter professional careers in the sciences, or other convergent problem-solving domains, this question is beyond the scope of the present study. Study 2 of the present research will correct for any potential confounds due to the gender imbalance of Study 1 by sampling a more even number of men and women. Nonetheless, gender differences in the occurrence of insights, or association between insight intensity and accuracy, remain an area ripe for future research.

## 5. Study 2 Introduction

Study 2 used a pre-registered, within-subjects design with established laboratory measures of convergent and divergent thinking. The primary objective was to explicitly examine divergent and convergent problem-solving contexts, as opposed to inferring them based on occupation. This allowed us to explore whether the diagnostic value of insights differs between these contexts, and whether such differences are consistent across people.

Convergent thinking was measured with the Compound Remote Associates Task (CRAT; Bowden & Jung-Beeman, 2003), in which individuals are presented with word triads (e.g., COTTAGE/SWISS/CAKE), and must find one word that can form a compound word with each (e.g., CHEESE). All problems in this task have a single, correct solution, making it one of the most widely accepted measures of convergent thinking in psychological research. Furthermore, past research has robustly demonstrated that solutions on the CRAT can either be reached through a sudden Aha! experience or through a more analytic strategy, in which potential words are systematically tested until a solution is found (e.g., Morrison et al., 2017; Moss & Cranford, 2012). Consistent with the pattern previously described, insight-based solutions are more likely to be accurate compared to analytic ones (Salvi et al., 2016). The CRAT therefore provides a well-established paradigm for indexing the insight–accuracy relationship.

To examine divergent thinking, the classic Alternate Uses Task (AUT; Guilford, 1967a) was used, in which individuals are asked to generate creative, unusual uses for everyday items (e.g., a brick). Unlike the CRAT, the AUT has no single, objective answer; instead, individuals are encouraged to generate many possible ideas with the goal of being maximally creative. The AUT is therefore an ideal complement for the CRAT, as both are widely accepted as measuring divergent and convergent creative cognition, respectively. In Study 2, both tasks used a trial-by-trial method, in which participants entered their responses one at a time, immediately followed by ratings of insight and idea quality. This design allowed us to reduce the temporal distance between idea generation and assessment relative to Study 1.

Study 2 extended and addressed several further limitations inherent to Study 1. First, objective idea quality scores could be computed from the creativity tasks, unlike in Study 1 where assessments of idea quality were measured solely with self-reports. For the AUT, the creative merit of each idea can be quantified by independent judges using consensual assessment techniques (Amabile, 1982), while participants’ total solution accuracy can be computed in the CRAT. These objective scores can then be compared to participants’ perceived quality of ideas, which was operationalized in parallel ways across the two tasks: in the divergent task, through self-rated creativity, and in the convergent task, by confidence in the accuracy of their solutions. The ability to compute both objective and subjective ratings of idea quality enables us to examine not only whether insights track with perceived quality, but whether this aligns with, or diverges from, the actual quality of the idea.

Second, Study 2 measured insight experiences on a continuous scale, allowing not only the presence but also the intensity of the insight experience to be captured. This reflects an important refinement from the binary measure used in Study 1 and supports a broader trend in insight research toward capturing more granular indices of the Aha! experience (Webb et al., 2016).

Third, Study 2 built from the motivational findings observed in Study 1 by examining whether similar patterns hold across divergent and convergent problem-solving contexts. After completing each type of creativity task, participants completed a series of questions to capture motivation, including feelings of inspiration and self-efficacy. These items closely paralleled those used in Study 1 but were completed immediately after each task rather than aggregated across the day. Once again, this shorter temporal distance between insight and its potential motivational correlates enables a tighter examination of the motivational signature of insight and how it may differ depending on problem-solving context.

Fourth, Study 2 included an individual difference measure of divergent thinking ability, which allowed us to begin to untangle whether potential divergences in the insight–accuracy relationship are driven by different types of *thinkers* or different types of *thinking*. Specifically, average objective creativity scores from the AUT were calculated as a measure of trait divergent thinking, which was then used to test whether the insight–accuracy relationship differs in strength, particularly for individuals who are high in divergent thinking. This approach allows us to test whether insight functions as a universal signal of solution quality, or whether its diagnostic value depends on the alignment between a person’s characteristic style of thinking and the cognitive demands of the task at hand.

These design features supported the following preregistered predictions. The primary prediction was that insight intensity would predict greater bias on the divergent thinking task, as measured by the difference between self-rated and objective creativity scores (H1). This prediction directly follows from the results of Study 1, in which writers were shown to overestimate the creative value of their ideas at the time of idea generation. Similarly, we predicted that insight intensity would predict self-rated creativity on the divergent thinking task (H2a) but show no association with objective creativity ratings (H2b). This hypothesis was, in part, based on unpublished pilot data which found no association between average insight intensity and objective creativity scores on the AUT between subjects. However, in contrast to the above-mentioned prediction, the pilot data showed an association between insight and creativity on a trial-by-trial basis—which was initially missed due to the non-significant between subjects effect. Next, replicating past research efforts, we hypothesized that insight intensity would predict solution accuracy on the convergent thinking task (H3a), as well as higher solution confidence (H3b). The inclusion of divergent thinking and convergent thinking in the same study enabled us to directly compare the strength of association between insight intensity and accuracy within convergent and divergent contexts, which we predicted would be stronger in the former as compared to the latter (H4). Finally, with respect to the potential motivational role of insight, we predicted that insight intensity would be associated with greater enjoyment, motivation, inspiration, meaningfulness, and self-efficacy across both the convergent and divergent creativity tasks (H5).

In addition to our primary hypotheses, two individual difference predictions were pre-registered as exploratory hypotheses. We sought to replicate the findings of Webb et al. (2021), which found that divergent thinking ability predicts insight intensity, but not accuracy, on a range of convergent thinking tasks. Thus, we predicted that objective creativity scores on the AUT would predict stronger insight intensity on the CRAT (H6) but show no association with solution accuracy on the CRAT (H7).

## 6. Study 2 Methods

### 6.1. Participants

Participants were recruited from the online platform Prolific (*N* = 108); all participants resided in the United States and were fluent in the English language. Data from eight participants were excluded due to poor response quality (*n* = 1) or failed attention checks (*n* = 7; see Exclusions below). This brought the final sample down to one hundred participants (60 female, 39 male, 1 non-binary), with a mean age of 21.02 (*SD* = 14.5). The majority of participants listed English as their primary language (*n* = 97, 97%) followed by Spanish and Chinese (both *n*s = 1, 2% total). Most participants reported college graduate as their highest level of educational attainment (*n* = 34, 34%), followed by high school diploma or equivalent (*n* = 24, 24%) and some college (*n* = 22, 22%).

### 6.2. Design

This study follows a cross-sectional, within-subjects design, in which all participants completed the Alternate Uses Task (AUT) and Compound Remote Associates Task (CRAT). The order of tasks was counterbalanced across participants.

### 6.3. Transparency Statement

All study methods, sample size, exclusion criteria, predictions, and analysis plan were pre-registered prior to data collection. Data were added to the registry following data collection. Sample size was computed using an a priori power analysis. We targeted a final sample of 100 participants and recruited 108 participants to allow for anticipated exclusions. The planned sample size provided approximately 80% power to detect correlations of r = 0.25 and over 90% power to detect correlations of r = 0.30, which were considered plausible effect sizes for the primary hypothesis.

### 6.4. Exclusions

Given data quality concerns in online samples, several pre-registered exclusion criteria were used to screen poor responders or bots. First, an attention check question was included in the survey, on which participants were asked, “Please move the slide to the extreme right (“More than 5 times”) to indicate you are paying attention.” Participants who did not respond accordingly were removed from data analysis (*n* = 7). Additionally, for analyses requiring within-person correlations, participants were excluded from the relevant analysis if they showed no variability in predictor or outcome on a given task. Thus, one person was excluded from data analysis, who had uniform responses across all insight and creativity ratings on the AUT. This brought the total number of participants down to one hundred (*N* = 100).

### 6.5. Divergent Thinking

Divergent thinking was assessed using the Alternate Uses Task (AUT; Guilford, 1967a). Participants were instructed to think of as many creative and novel ways as possible to use a series of commonplace objects (e.g., a brick, a tin can). Participants were forced to enter 5 ideas per object, with a total of three objects: brick, tin can, and cardboard box, presented in randomized order. The AUT was scored by two blind, independent raters on a scale of 1–5 (*not creative* to *highly creative*), and a 0 given to nonsensical or invalid responses. After confirming sufficient inter-rater reliability (see Scoring section below), scores were averaged across both raters to compute an “objective” creativity score for each idea. Trial-by-trial analysis used this variable as a measure of idea quality in the divergent problem-solving context. Analyses involving average objective creativity additionally averaged the two raters’ scores across all ideas, with the averaged scores ranging from 1 to 5.

### 6.6. Convergent Thinking

Convergent thinking was assessed using an adapted version of the Compound Remote Associates Task (CRAT; Bowden & Jung-Beeman, 2003); the stimulus set used for this study was taken from Zedelius and Schooler (2015). The set includes 30 problems, in which participants are shown three stimulus words on the screen (e.g., BOARD/MAGIC/DEATH) with a text box below. Participants are instructed to generate a fourth word that forms a compound word or phrase with each of the stimulus words (e.g., BLACK), and are given a total of 30 s to solve each problem. If they do not know the answer, they are instructed to write a best guess. Each solution was coded either 0 for incorrect, or 1 for correct, with non-responses excluded from analyses.

### 6.7. Insight

After each CRAT solution and each AUT idea, participants rated the strength of any insight experience they had on a continuous sliding scale from 0 (*no insight*) to 10 (*very strong insight*). Participants were provided with the same definition of insight for both tasks—described as ideas that appear abruptly and are somewhat surprising, and were contrasted with ideas that emerge gradually or systematically by brainstorming.

### 6.8. Subjective Idea Quality

Subjective idea quality was operationalized as self-reported confidence in the accuracy of provided solutions in the CRAT, and self-reported creativity in the AUT. After each CRAT trial, participants rated their confidence that their solution was correct on a 5-point scale (1 = *not at all confident*, 5 = *very confident*). After generating each idea on the AUT, participants rated the creativity of their idea on a 5-point scale (1 = *not at all creative*, 5 = *extremely creative*).

### 6.9. Post-Task Questions

To capture feelings of motivation, after each task (i.e., the CRAT and AUT), participants responded to single-item questions capturing: how much they enjoyed the activity, how confident they felt in their ability to generate creative ideas/solve word problems (for the AUT and CRAT, respectively), how motivated they felt during the activity, as well as how meaningful and inspiring they found the activity. Each item was rated on a Likert scale from 1 to 5.

### 6.10. Procedures

After providing informed consent, participants were either presented with instructions for the AUT or CRAT first, with task presentation counterbalanced across participants. After reading through the task instructions, participants were provided with the definition of an Aha! experience, or insight moment, and how it differs from non-Ahas. They were then informed that, following each solution or idea they generated, they would be asked to indicate the intensity of insight they experienced, if any. Ideas in the AUT were entered one at a time, followed by the insight and self-rated creativity (1–5 scale; not creative to very creative) questions. Solutions in the CRAT were similarly rated, immediately following their generation, on insight and confidence in the accuracy of the provided solution. Following both the CRAT and AUT, participants completed the post-task questions to capture feelings of motivation. Finally, participants completed basic demographics questions and were compensated for their time.

### 6.11. Data Scoring

#### Alternate Uses Task

Two independent raters, blind to condition, rated the ideas generated for the AUT on their creative merit. Inter-rater reliability demonstrated good to excellent reliability across prompts: ICC(2,1) = 0.80 for tin can, 0.73 for brick, and 0.67 for cardboard box; pooled ICC(2,1) across all prompts = 0.74. The two judges’ ratings were averaged to form a composite creativity score for each idea, with ICC(2,1) = 0.85 across the pooled sample, indicating excellent reliability of the composite measure used in subsequent trial-by-trial analyses. For the individual differences analyses (where AUT performance was operationalized as a single per-participant variable), rater-averaged ratings were then further averaged across ideas and objects to form a single composite creativity score per participant.

### 6.12. Analytic Strategy

To account for the hierarchical nature of the data, in which multiple trials (Level 1) are nested within participants (Level 2), mixed modeling techniques were used for the primary analyses. The predictors included person-mean-centered insight intensity and person means of insight intensity, which allow us to separate the within-person and between-person effects of insight intensity on our outcomes. Participants were included as a random effect (intercepts only).

For the CRAT solution accuracy analyses, in which the DV is a binary (1 = correct, 0 = incorrect), a generalized linear mixed-effects model was fitted with a binomial error distribution and a logit link function using maximum likelihood estimation (Laplace approximation). The model was estimated in R (version 2024.04.1) using the *lme4* package version 1.1.36 (Bates et al., 2015). Effect sizes are reported as odds ratios (*OR*) with 95% confidence intervals, along with marginal and conditional *R*^2^ (Nakagawa & Schielzeth, 2013) for overall model fit.

For all other analyses, in which the DV is continuous (e.g., self-rated creativity, objective creativity, solution confidence), linear mixed-effects models were fit using the *lme4* R package (Bates et al., 2015). The models used restricted maximum likelihood (REML) estimation, with Satterthwaite approximations for degrees of freedom and *p*-values provided by the *lmerTest* package version 3.1.3 (Kuznetsova et al., 2017). Effect sizes are reported as unstandardized regression coefficients (*b*) with 95% confidence intervals, along with semi-partial *R*^2^ with bootstrapped CIs (1000 iterations; Stoffel et al., 2021) for individual fixed effects and marginal and conditional *R*^2^ for overall model fit.

## 7. Results

### 7.1. H1: Insight Intensity and Bias on the AUT

Our first prediction was that insight intensity would predict greater bias on the AUT. A bias score was calculated for each AUT trial by subtracting objective creativity ratings from self-rated creativity scores such that positive scores indicate a greater tendency to self-rate ideas as more creative than objective raters. Then, a linear mixed effects model was fitted with bias as the outcome variable. The model revealed a significant within-persons effect of insight intensity on bias scores, such that on trials where people experienced stronger insights, they also tended to rate their ideas as more creative than objective raters, *b* = 0.19, *SE* = 0.014, *t*(1495) = 13.06, *p* < .001, 95% CI [0.16, 0.22], partial  $R^{2}$  = 0.064. Additionally, the model revealed a significant between-person effect of insight intensity on bias scores, such that people who experienced stronger insights on the AUT overall tended to rate their ideas as more creative than objective, trained raters, *b* = 0.31, *SE* = 0.031, *t*(1495) = 10.25, *p* < .001, 95% CI [0.25, 0.37], partial  $R^{2}$  = 0.21. Overall, the fixed effects explained 27% of the variance in bias scores (marginal  $R^{2}$  = 0.27), with the full model explaining 44% (conditional  $R^{2}$  = 0.44). Here, we find evidence to suggest that stronger insight experiences lead people to overestimate the creativity of their ideas in a divergent thinking context.

### 7.2. H2a: Insight Intensity and Self-Rated Creativity on the AUT

The second preregistered prediction was that insight intensity would predict self-rated creativity on the AUT. To test this hypothesis, a linear mixed effects model was fitted with subjective creativity scores as the outcome variable. Consistent with our hypothesis, the model revealed a significant within-person effect of insight intensity on self-rated creativity: on trials where participants report greater insight intensity, they tended to rate their ideas as more creative, *b* = 0.27, *SE* = 0.01, *t*(1495) = 25.50, *p* < .001, 95% CI [0.25, 0.29], partial  $R^{2}$  = 0.17. Additionally, there was a significant between-person effect of insight intensity on self-rated creativity, such that people who experience, on average, more intense insights on the AUT tended to rate their ideas as more creative, *b* = 0.33, *SE* = 0.02, *t*(1495) = 14.13, *p* < .001, 95% CI [0.29, 0.38], partial  $R^{2}$  = 0.32. Overall, the fixed effects explained 48% of the variance in self-rated creativity scores, (marginal  $R^{2}$  = 0.48), with the full model explaining 62% (conditional  $R^{2}$  = 0.62).

### 7.3. H2b: Insight Intensity and Objective Creativity on the AUT

The next pre-registered prediction was that insight intensity would *not* be significantly associated with objective (rather-judged) creativity—or idea quality—on the AUT. To test this hypothesis, a linear mixed effects model was fitted with objective creativity scores as the outcome variable. In contrast to our predictions, the model revealed a significant within-person effect of insight intensity on objective creativity, such that on trials where participants reported greater insight intensity, they also received higher objective creativity scores, *b* = 0.08, *SE* = 0.01, *t*(1495) = 5.76, *p* < .001, 95% CI [0.05, 0.10]. However, the model revealed that the between-persons effect of insight intensity on objective creativity was non-significant, such that people who reported stronger insights on the AUT did not tend to have higher objective creativity scores, *b* = 0.02, *SE* = 0.02, *t*(1495) = 0.97, *p* = 0.33, 95% CI [−0.02, 0.06]. Overall, the fixed effects explained 2.1% of the variance in objective creativity scores, (marginal  $R^{2}$  = 0.021), with the full model explaining 15% (conditional  $R^{2}$  = 0.15).

### 7.4. H3a: Insight Intensity and Solution Accuracy on the CRAT

Our fourth pre-registered prediction was that insight intensity on the CRAT would predict solution accuracy. In line with our prediction, the model revealed a significant within-person effect of insight intensity on solution accuracy, *b* = 0.65, *SE* = 0.03, *z* = 25.11, *p* < .001, 95% CI [0.60, 0.71], OR = 1.92, 95% CI [1.82, 2.03]. On trials where participants reported greater insight intensity, they were more likely to provide an accurate solution to the CRAT problem. Additionally, there was a significant between-person effect of insight intensity on solution accuracy, such that individuals who reported greater insights were more likely to respond correctly on the CRAT overall, *b* = 0.13, *SE* = 0.06, *z* = 1.99, *p* = .046, 95% CI [0.002, 0.25], OR = 1.13, 95% CI [1.01, 1.27]. Overall, the fixed effects explained 38% of the variance in solution accuracy (marginal  $R^{2}$  = 0.38), with the full model explaining 51% (conditional  $R^{2}$  = 0.51).

### 7.5. H3b: Insight Intensity and Solution Confidence on the CRAT

In line with our fifth pre-registered prediction, there was also a significant within-person effect of insight intensity on solution confidence, such that on trials where participants reported more intense insights, they also were more confident in the accuracy of their solutions, *b* = 0.41, *SE* = 0.0055, *t*(2995) = 75.45, *p* < .001, 95% CI [0.40, 0.42], partial  $R^{2}$  = 0.52. The between-person effect of insight intensity on solution confidence was also significant, *b* = 0.29, *SE* = 0.02, *t*(2995) = 11.84, *p* < .001, 95% CI [0.24, 0.34], partial  $R^{2}$  = 0.12: people who experienced more intense insights were more confident in the accuracy of their solutions on the CRAT overall. Overall, the fixed effects explained 64% of the variance in solution confidence (marginal  $R^{2}$  = 0.66), with the full model explaining 72% (conditional  $R^{2}$  = 0.72).

### 7.6. H4: Insight-Accuracy Relationship on the CRAT vs. AUT

Our sixth preregistered prediction was that the within-person, insight–accuracy association would be stronger on the CRAT (convergent task) as compared to the AUT (divergent thinking task). To test this, we adopted a calibration-based approach drawn from the metacognition literature (Nelson, 1984; Fleming & Lau, 2014). For each participant, we computed two within-person rank correlations between insight intensity and task performance: one for CRAT trials (insight intensity correlated with solution accuracy, point-biserial in form but computed as Spearman’s ρ for consistency) and one for AUT trials (insight intensity correlated with objective creativity ratings, Spearman’s ρ). This yielded two calibration scores per participant, each indexing how well their trial-level insight experience tracks objective performance on that task. Correlations were then computed, and applied separately to each participant’s trial-level data within each task.

Calibration scores were Fisher *z*-transformed to normalize their distribution, and the two scores were compared within participants using a paired-samples *t*-test. The test revealed a significant difference in the Fisher *z*-transformed calibration scores for the CRAT and AUT, *mean difference* = 0.52, *p* < .001, 95% CI [0.41, 0.63]. Examination of the unstandardized, mean rho coefficients across both tasks revealed that CRAT calibration scores (mean ρ = 0.59,  $\rho^{2}$  = 0.35) were higher than AUT calibration scores (mean ρ = 0.16,  $\rho^{2}$  = 0.03), accounting for 35% and 3% of the variance in scores, respectively. This finding suggests that the within-person association between insight intensity and task performance was stronger for the CRAT compared to the AUT.

### 7.7. H5: Insights and Motivation

The last set of analyses, preregistered as primary analyses, explore whether insight intensity predicts higher measures of task motivation on both the convergent and divergent creativity tasks. To test this hypothesis, bivariate correlations were computed between insight intensity and each of the five task motivation outcomes (enjoyment, motivation, inspiration, meaningfulness, and self-efficacy), separately for the convergent (CRAT) and divergent (AUT) tasks.

Bivariate correlations revealed that average insight intensity on the AUT was positively correlated with each of the motivational task measures—enjoyment, motivation, meaningfulness, inspiration, self-efficacy, and mood (all *p*s < .01; see Table 2).

Similarly, average insight intensity on the CRAT was significant and positively correlated with each motivational measure (all *p*s < .01; all see Table 3). In sum, these findings suggest that insight experiences play an adaptive role in creative motivation across both convergent and divergent thinking tasks.

### 7.8. Exploratory Analyses

To examine the potential role of individual differences in the association between insight and accuracy, two preregistered exploratory analyses were conducted.

#### 7.8.1. H6: Individual Differences in Divergent Thinking and Insight Intensity on the AUT

First, we anticipated that objective creativity scores on the divergent thinking task would predict greater insight intensity on the convergent thinking task—a finding that is consistent with prior research (Webb et al., 2021). To test this, we computed a single Pearson correlation between each participant’s average objective creativity score on the AUT and their average insight intensity on the CRAT. Both variables were averaged across trials within each task to yield one score per participant per task. The test revealed a significant correlation between objective creativity scores on the AUT and average insight intensity on the CRAT, *r* = 0.27, *p* = .007, 95% CI [0.075, 0.44]. People who received higher creativity scores on the AUT tended to experience stronger insights during the CRAT. This finding replicates the results of Webb et al. (2021), and suggests that divergent thinking ability predicts stronger insight phenomenology on convergent thinking tasks.

#### 7.8.2. H7: Individual Differences in Divergent Thinking and Accuracy on the CRAT

We also predicted that objective creativity scores on the divergent thinking task would not be associated with solution accuracy on the CRAT—again in line with the same prior research (Webb et al., 2021). To test this, we computed a Pearson correlation between each participant’s average objective creativity score on the AUT and their proportion of correct solutions on the CRAT, with both variables averaged across trials within each task to yield one score per participant per task. In line with past research, the correlation between average objective creativity scores and accuracy on the CRAT was non-significant, *r* = 0.095, *p* = 0.35, 95% CI [−0.104, 0.29].

Because a non-significant correlation alone is not sufficient to support a null claim, we additionally conducted a two one-sided tests (TOST) equivalence test, with the smallest effect size of interest set at r = ±0.10. Support for this hypothesis was preregistered as requiring statistical equivalence to zero (within ±0.10) in the TOST procedure. The TOST equivalence test was non-significant, *p* = 0.48, indicating that neither a meaningful effect nor equivalence to zero could be established. Thus, we do not have sufficient evidence to conclude that performance on the AUT (divergent thinking task) does not predict accuracy on the CRAT (convergent thinking task).

## 8. Interim Discussion—Study 2

Study 2 expanded upon the central finding of Study 1, which offered initial evidence that the reliability of insights—as an indicator of idea quality—may critically depend upon the problem-solving context in which the ideas are generated. Using a within-subjects design, in which all participants completed both a convergent and divergent thinking task, we directly compared the diagnostic value of insights across problem-solving contexts, while also avoiding potential individual difference confounds associated with profession in Study 1.

The results of Study 2 were largely consistent with our preregistered predictions. First, insight intensity was a reliable predictor of idea quality on the CRAT, both on a trial-by-trial basis and at an individual difference level. Insight intensity predicted both objective solution accuracy, replicating the well-established insight–accuracy relationship previously observed in convergent thinking tasks (e.g., Salvi et al., 2016; Laukkonen et al., 2023), as well as self-reported solution confidence. Thus, Study 2 provides further support for the Eureka Heuristic theory, as the results demonstrate that insights serve as a reliable signal of accuracy on convergent thinking problems (Laukkonen et al., 2023).

The association between insight intensity and idea quality on the divergent thinking task diverged slightly from our predictions. While the predicted relationship was observed between insight intensity and self-rated creativity on the AUT, a relationship between insight intensity and objective creativity was also observed. Although this latter association was not anticipated, it aligns with results from an unpublished pilot study where we found a significant within-person effect of insight intensity on objective creativity scores; however, this finding was initially missed, as the association between average insight intensity and objective creativity at the person level was first examined and found to be non-significant. This discrepancy highlights the importance of examining trial-by-trial effects in divergent thinking to fully capture the nature of the relationship between insights and performance.

That said, the effect of insight intensity on performance was quite modest. Insight intensity accounted for only 2% of the variance in objective creativity and was observed only at the within-person level; on the other hand, individuals who experience more intense insights overall do not demonstrate correspondingly more creativity. The significant within-person relationship between divergent thinking performance and insight intensity is nevertheless theoretically meaningful and aligns with the pattern of results found in the Gable et al. (2019) data from Study 1; namely, that writers rated their Aha! ideas as more creative than non-Aha ideas at the time of idea generation—an effect that became marginal at 6 months follow up. Furthermore, another recent study also found that insight experiences predict creativity and novelty of ideas on a divergent thinking task (Smith et al., 2026). Taken together, these findings suggest that insight experiences can provide some indication of idea quality in divergent thinking contexts.

Critically, the diagnostic value of insights in divergent contexts is far outweighed by their inflationary effect on perceived creativity. In line with our preregistered predictions, insight intensity predicted bias on the AUT, or a greater tendency to overestimate the creativity of ideas at the time of idea generation; this effect was observed both on a trial-by-trial basis and at the person level. The disproportion between the modest diagnostic value of insight and its substantial inflationary effect is perhaps most clearly captured by the calibration analysis. The calibration scores reflect the degree to which the intensity of the insight experience tracks the objective idea quality on a trial-by-trial basis, within persons. Our findings revealed that the strength of association between insight intensity and accuracy was significantly higher on the CRAT compared to the AUT. Indeed, within the same individuals, the strength of the insight–accuracy relationship was nearly four times stronger in the convergent context compared to the divergent context, underscoring the general pattern that the diagnostic value of insights is substantially greater in the former context.

Individual differences might also play an important role in the insight–accuracy association, such that individuals who are more prone to experiencing insights overall might be predisposed to false insights (Webb et al., 2019). Indeed, Study 2 replicated past findings that divergent thinking ability on the AUT predicts insight intensity on the CRAT but not solution accuracy (Webb et al., 2021). However, we did not find definitive evidence for the null hypothesis, and thus cannot conclude that divergent thinking is unrelated to accuracy on convergent problems. Still, this replication provides suggestive evidence that people high in divergent thinking might be prone to experiencing more intense insights, without a corresponding gain in the diagnostic value of those insights—at least in convergent problem-solving contexts. To the extent that this dissociation generalizes, it suggests that the *Aha! glow* observed in divergent thinking problems might be amplified among precisely the individuals who are most likely to work in such contexts. The writers in Study 1, for example, were not only working in a divergent domain, but were, by selection, divergent thinkers. In the Supplementary Materials, Gable et al. (2019) found that writers were more likely to report an Aha! compared to physicists, aligning with the broader pattern that divergent thinkers are more likely to experience insight phenomenology. As the results from Study 2 suggest, such individuals might also experience particularly strong insights, without a corresponding increase in calibration—a trend that would explain the inflated creativity ratings writers had at the time of idea generation.

Finally, consistent with our predictions, we found that insight intensity was positively correlated with several self-reported features of task motivation—enjoyment, self-confidence, motivation, meaning, inspiration, and mood—across both tasks. The pattern of results aligns with those observed in Study 1 and suggests that Aha! experiences might play an adaptive role in creative motivation (see General Discussion for a more detailed discussion of this proposal). Additionally, consistent with the findings in Study 1, the strength of the correlations was broadly comparable across the CRAT and AUT; thus, the motivational role of insights appears to generalize across contexts, in contrast to the epistemic function of insight, which appears more context-dependent.

## 9. General Discussion

The present research explored whether insights act as a reliable marker of idea quality across convergent and divergent problem-solving contexts. To test this question, Study 1 examined daily diary data from two professional domains—professional writers and physicists—as real-world proxies for divergent and convergent problem-solving contexts, while Study 2 employed a controlled, within-subjects design with laboratory measures of convergent and divergent thinking. Across both studies, we find a similar pattern; the diagnostic value of insights appears to be reliable in convergent contexts but systematically overweighted in divergent contexts.

These findings provide an important extension to the Eureka Heuristic, which proposes that insights serve an epistemic role by pointing us towards solutions that are likely to be useful or true. First, the present research adds further support for the Eureka Heuristic in both real-world and laboratory contexts for convergent thinking. In Study 1, the Aha! ideas of physicists were rated as more creative than non-Aha ideas at the time of idea generation and were found to become even more valuable over time. Study 2 found that insight intensity functioned as a reliable indicator of solution quality on the CRAT, predicting both self-perceived accuracy (i.e., confidence) and objective accuracy. In these cases, the role of the insight appears to be consistent with that proposed in the Eureka Heuristic, namely, acting as a metacognitive signal of idea quality.

However, we also uncovered an important boundary condition to the Eureka Heuristic; the Aha! experience appears to bias perceptions of idea quality within divergent thinking contexts. In Study 1, professional writers—who typically work on divergent creativity problems—were found to systematically overestimate the creativity of their Aha! ideas at the time of generation, as indicated by a decrease in the perceived creativity of those same ideas 6 months later. In Study 2, we discovered a parallel pattern, such that creative responses were reliably overweighted relative to objective creativity scores. While the intensity of insights predicted both subjective and objective creativity scores, the subjective scores far overshadowed the confidence the ideas merit based on the objective scores. Thus, while insights may track the relative creative value of divergent ideas, at least to a modest degree, the phenomenological impact of the insight creates an *Aha! glow*, which leads to a systematic overestimation of an idea’s actual creative merit.

Two further pieces of evidence from Study 2 add support to this assertion. First, we found that insight intensity predicted bias, or a greater tendency to rate one’s own ideas as more creative than objective raters, on the alternate uses task. In other words, it appears that strong insights can inflate our perception of idea quality in divergent thinking contexts. Our results further showed that the association between insight intensity and accuracy was nearly four times stronger for the CRAT compared to the AUT, accounting for 35% versus 3% of the variance in task performances, respectively. This indicates that insights are substantially better calibrated to solution accuracy in convergent thinking contexts.

Taken together, these results provide the first evidence that insight experiences produce an *Aha! glow*, which is heightened among divergent thinking contexts; when a novel creative idea initially arises, the phenomenological experience of the insight inflates perceptions of idea quality, rendering insights less reliable as epistemic signals. The *Aha! glow* phenomenon may have implications that extend beyond idea generation. As previously noted, past research suggests the existence of an “insight bias,” or lay belief by which people overestimate the value of insight solutions and underestimate the role of slow, effortful reasoning during creative idea generation (Lucas & Nordgren, 2022). The present research adds to this work in two ways. First, it suggests a possible developmental pathway for this “insight bias;” if individuals repeatedly experience the *Aha! glow*—an inflation in the perceived quality of their insight-based ideas at the moment of idea generation—they may, over time, develop enduring beliefs that Aha! ideas are more integral to creative thinking. Secondly, our findings show that the lay belief itself does not hold in divergent contexts. Even though the Aha! ideas of writers were rated more creative than non-Aha ideas early on, over time this gap narrowed such that by six-month follow-up the two types of ideas were rated similarly. Put another way: though people believe creativity is best supported by flashes of insight, the ideas they come to value most over time appear only modestly more likely to have arrived with an Aha! than without one—at least in divergent contexts.

Despite leading to a systematic overestimation of perceived creativity in divergent contexts, we propose that the *Aha! glow* may serve an important role in the creative process. Specifically, this very inflation may provide the motivational impetus necessary to pursue ideas whose ultimate value can only be fully realized through sustained development and refinement. Given that the goal of divergent thinking is the generation of genuinely novel ideas, it is rarely possible to determine whether an idea will prove paradigm-shifting or misguided until it is fully fleshed out and evaluated within its context. Divergent thinkers must therefore act on something of a leap of faith, continuing to explore novel ideas despite uncertainty about the eventual payoff. What appears, from an epistemic standpoint, like overconfidence may, from a motivational standpoint, provide the necessary drive and prolonged engagement on which creative outputs often depend. Consistent with this possibility, we found that insights were associated with greater self-report measures of creative motivation and self-efficacy—both between persons (Study 1) and across tasks (Study 2).

This potentially adaptive value of the *Aha! glow* resembles the concept of epistemic innocence, which refers to a cognition or belief that carries an epistemic cost, but also brings an epistemic benefit to the agent that could not be conferred by any other belief available to the agent at that time (Bortolotti, 2015). This idea was initially coined in reference to motivated delusions, which, although epistemically false, might help to preserve an agent’s epistemic functionality in the face of overwhelmingly negative emotions or incoherent experiences. However, Bortolotti (2015) notes that other types of cognition may be considered epistemically innocent, including self-deception and distorted memory. The *Aha! glow* might constitute another such example, as although strong insight phenomenology bears the epistemic cost of overconfidence, it may also confer an epistemic benefit by motivating individuals to continue generating creative ideas. Over time, this creative pursuit can lead to the discovery of novel and useful products.

The present findings are also consistent with recent theorizing which suggests that insights may act as positive reinforcement in creative and aesthetic contexts, a process termed the *Discovery Feedback Loop* (Gross, in press). According to this account, certain contexts provide greater opportunity for insight experiences, such as creative problem solving and artistic engagement. These insight experiences, in turn, promote feelings of pleasure and drive, which reinforces further engagement with these behaviors and contexts. Over time, this prolonged engagement scaffolds the development of stable individual differences in creative skill and output which increases, further still, the likelihood of engagement in these contexts. In short, the positively reinforcing nature of insights may support both short-term creative engagement on a given problem, and longer-term developmental outcomes in creative skill and aptitude.

### Limitations and Future Directions

There are several limitations in the present research. First, the primary outcome variable in Study 1 was subjective creativity ratings, which may not reflect the true quality of ideas for writers and physicists. Future studies investigating real-world Aha! ideas could relate self-ratings to external standards of value, such as the ratings from independent experts in each field, to further examine insight–accuracy relationships within divergent and convergent professional domains. Similarly, across both studies, creative motivation was captured using self-report responses, which are susceptible to individual bias or meta-cognitive inaccuracies. Future research could extend the present findings by exploring whether insights predict behavioral indices of creative motivation, such as active choices to engage in creative behaviors. As the motivational analyses in the present research were correlational, we were also not able to establish a direction of causality. Though our primary interpretation is that insights promote feelings of motivation, it is possible that people higher in creative motivation are more likely to experience insights. Future studies could clarify the relationship between insights and motivation by using an experimental design in which the strength of insights is manipulated and downstream effects on motivational outcomes are measured.

A further consideration is how insight intensity was measured in Study 2; rather than asking participants to categorize their ideas as either insight or analytic, participants rated the intensity of their insights on a continuous scale from 0 (*no insight*) to 10 (*very strong insight*). This more granular measure of insight is potentially better equipped to capture the “real” signature of insight, which may be experienced even during ostensibly analytic solutions. In other words, rather than a discrete line between insight-based solutions and analytic-based solutions, insights may reflect a more graded phenomenology that can accompany even smaller, incremental steps in the creative process, albeit with weaker intensity. Future research should continue to explore this possibility, including investigating whether insights must reach a certain threshold of felt intensity to reliably signal idea quality.

Another concern is the assumption that the internal experience of insight remains constant across both convergent and divergent problem-solving contexts. The present research treats divergent and convergent insights as phenomenologically identical, using the same definition of insight and a common measurement scale across both problem types. Notably, insight intensity showed similar correlational patterns with motivational variables across both problem contexts in Study 2, which provides initial evidence that Aha! experiences share a common experiential core. However, we cannot rule out the possibility that the internal experience of insight differed in ways that were not captured by our measures. Future research could utilize multi-dimensional measures of insight (Danek & Wiley, 2017), to further explore whether insight experiences are phenomenologically similar across divergent and convergent creativity contexts. Furthermore, should there exist substantial differences in the internal experience of insight between problem types, this could provide a mechanistic explanation for the dissociation in the diagnostic value of insight reported in the present research.

Another important concern is that insights may be less diagnostic in divergent contexts because the measure of objective creativity is less reliable than measures of idea accuracy in convergent thinking contexts. The Consensual Assessment Technique (Amabile, 1982) used in this study is considered the gold standard approach for evaluating creativity on divergent thinking tasks. Prior creativity research treats the performance scores of two independent raters as capturing “objective” individual differences in creativity (Dollinger & Shafran, 2005; Miller & Tal, 2007; Barth & Stadtmann, 2021). Nevertheless, the use of raters for determining creative idea quality is unique to divergent contexts, and likely introduces some level of subjectivity as compared to convergent scores in which there is a single, well-defined solution. It is therefore possible that the diagnostic function of insight might only hold for problems which have one clearly defined solution that serves as a standard of quality for people to evaluate their given ideas.

Finally, it is also important to note that across both studies, participants were sampled from the United States, and most people reported English as their primary language. Thus, while cultural differences in insight phenomenology might exist, the common geographical origin of participants in the present research does not allow for a proper examination of this question. Future studies could recruit participants from multiple countries and replicate the present research to explore whether the same finding of an *Aha! glow* for divergent creativity contexts extends to a more geographically diverse sample.

The present research provides the first evidence that insights are a less reliable diagnostic signal in divergent thinking contexts. Thus, future research could replicate this finding across different divergent thinking tasks and explore potential mechanisms which underlie the *Aha! glow*. For example, future studies might explore how different dimensions of insight phenomenology (e.g., suddenness, pleasure, certainty) predict bias on divergent thinking tasks.

## 10. Conclusions

To our knowledge, this is the first study to show a breakdown of the insight–accuracy association among divergent thinking contexts. Across two studies, we find converging evidence for an *Aha! glow*, or consistent pattern in which insights predict inflated perceptions of idea quality in divergent thinking contexts, including: (1) a systematic decline in self-reported creativity over time for writers, but not physicists; (2) a robust association between insight intensity and bias on a laboratory measure of divergent thinking; (3) a nearly fourfold stronger calibration between insight intensity and solution quality on convergent compared to divergent thinking tasks; and (4) an individual difference bias such that divergent thinkers tend to experience stronger insights but not more accuracy on convergent thinking tasks. Although Aha!’s may imbue divergent ideas with an unjustified sense of merit, this inflation may itself be of value, as it is associated with a sense of meaning, motivation and inspiration. Writers, artists, and the many other individuals who toil over long periods on creative projects, may depend on the welcome boost of the *Aha! glow*, even if it is exaggerated.

## Figures and Tables

**Table 1:** Estimated Marginal Means for Creativity Ratings by Profession, Idea Type, and Time.

Profession	Idea Type	Time	*M*	*SE*	*n*	95% CI
Physicists	Non-aha	T1	3.16	0.17	[198]	[2.83, 3.49]
Physicists	Non-aha	T2	3.80	0.17	[185]	[3.47, 4.14]
Physicists	Aha	T1	3.98	0.19	[108]	[3.60, 4.35]
Physicists	Aha	T2	4.45	0.20	[100]	[4.07, 4.84]
Writers	Non-aha	T1	3.95	0.16	[217]	[3.65, 4.26]
Writers	Non-aha	T2	4.33	0.16	[212]	[4.02, 4.64]
Writers	Aha	T1	5.07	0.16	[217]	[4.76, 5.37]
Writers	Aha	T2	4.59	0.16	[208]	[4.28, 4.90]

Note. Means, standard errors, and confidence intervals are estimated marginal means from the linear mixed-effects model. *n* = the number of observations per cell. Creativity ratings were made on a 1–5 scale.

**Table 2:** Pearson Correlations Among AUT Variables.

Variable	1	2	3	4	5	6	7
1. Insight Intensity (mean)	—						
2. Enjoyment	0.29 **	—					
3. Confidence	0.53 ***	0.63 ***	—				
4. Motivation	0.31 **	0.63 ***	0.45 ***	—			
5. Meaning	0.34 ***	0.72 ***	0.53 ***	0.66 ***	—		
6. Inspiration	0.43 ***	0.77 ***	0.60 ***	0.77 ***	0.78 ***	—	
7. Mood	0.29 **	0.71 ***	0.51 ***	0.70 ***	0.62 ***	0.69 ***	—

Note. ** *p* < .01. *** *p* < .001. *N* = 100.

**Table 3:** Pearson Correlations Among CRAT Variables.

Variable	1	2	3	4	5	6	7
1. Insight Intensity (mean)	—						
2. Enjoyment	0.46 ***	—					
3. Confidence	0.50 ***	0.50 ***	—				
4. Motivation	0.33 ***	0.66 ***	0.40 ***	—			
5. Meaning	0.30 **	0.62 ***	0.40 ***	0.57 ***	—		
6. Inspiration	0.32 **	0.61 ***	0.44 ***	0.57 ***	0.72 ***	—	
7. Mood	0.29 **	0.74 ***	0.33 ***	0.61 ***	0.54 ***	0.60 ***	—

Note. ** *p* < .01. *** *p* < .001. *N* = 100.

## Data Availability

Data supporting reported results can be found here: https://doi.org/10.17605/OSF.IO/RS23W.

## Supplementary Materials

The following supporting information can be downloaded at: https://www.mdpi.com/article/10.3390/jintelligence14080160/s1, Table S1: Type III Tests of Fixed Effects from the Profession × Idea Type × Time Linear Mixed-Effects Model on Creativity Ratings.

## Abbreviations

The following abbreviations are used in this manuscript:

CRAT	Compound Remote Associates Task
AUT	Alternate Uses Task

## References

[B1-jintelligence-14-00160] Amabile T. M. (1982). Social psychology of creativity: A consensual assessment technique. Journal of Personality and Social Psychology.

[B2-jintelligence-14-00160] Barth P., Stadtmann G. (2021). Creativity assessment over time: Examining the reliability of CAT ratings. The Journal of Creative Behavior.

[B3-jintelligence-14-00160] Bates D., Mächler M., Bolker B., Walker S. (2015). Fitting linear mixed-effects models using lme4. Journal of Statistical Software.

[B4-jintelligence-14-00160] Bortolotti L. (2015). The epistemic innocence of motivated delusions. Consciousness and Cognition.

[B5-jintelligence-14-00160] Bowden E. M., Jung-Beeman M. (2003). Normative data for 144 compound remote associate problems. Behavior Research Methods, Instruments, & Computers.

[B6-jintelligence-14-00160] Carlisle N. A., Simonsson O., MacCarthy S., Elliott M. N., Miller G. H., Goldberg S. B., Shallcross R. A., Hendricks P. S. (2026). Prevalence, types, and demographic characteristics associated with major life changes following psychedelic use. Scientific Reports.

[B7-jintelligence-14-00160] Danek A. H., Wiley J. (2017). What about false insights? Deconstructing the Aha! experience along its multiple dimensions for correct and incorrect solutions separately. Frontiers in Psychology.

[B8-jintelligence-14-00160] Dollinger S. J., Shafran M. (2005). Note on consensual assessment technique in creativity research. Perceptual and Motor Skills.

[B9-jintelligence-14-00160] Fleming S. M., Lau H. C. (2014). How to measure metacognition. Frontiers in Human Neuroscience.

[B10-jintelligence-14-00160] Gable S. L., Hopper E. A., Schooler J. W. (2019). When the muses strike: Creative ideas of physicists and writers routinely occur during mind wandering. Psychological Science.

[B11-jintelligence-14-00160] Gross M. E., Omigie D., Frascaroli J., Contesi F. (in press). The discovery feedback loop: How insights sustain creative engagement across time. Arts and the drive for knowledge: Philosophical, psychological and neuroscientific perspectives.

[B12-jintelligence-14-00160] Guilford J. P. (1967a). Creativity: Yesterday, today and tomorrow. The Journal of Creative Behavior.

[B13-jintelligence-14-00160] Guilford J. P. (1967b). The nature of human intelligence.

[B14-jintelligence-14-00160] Hedne M. R., Norman E., Metcalfe J. (2016). Intuitive feelings of warmth and confidence in insight and noninsight problem solving of magic tricks. Frontiers in Psychology.

[B15-jintelligence-14-00160] Kuhn T. S. (1962). The structure of scientific revolutions.

[B16-jintelligence-14-00160] Kuznetsova A., Brockhoff P. B., Christensen R. H. B. (2017). lmerTest package: Tests in linear mixed effects models. Journal of Statistical Software.

[B17-jintelligence-14-00160] Laukkonen R. E., Kaveladze B. T., Tangen J. M., Schooler J. W. (2020). The dark side of Eureka: Artificially induced Aha moments make facts feel true. Cognition.

[B18-jintelligence-14-00160] Laukkonen R. E., Webb M., Salvi C., Tangen J. M., Slagter H. A., Schooler J. W. (2023). Insight and the selection of ideas. Neuroscience and Biobehavioral Reviews.

[B19-jintelligence-14-00160] Lucas B. J., Nordgren L. F. (2022). Lay people’s beliefs about creativity: Evidence for an insight bias. Trends in Cognitive Sciences.

[B20-jintelligence-14-00160] McGovern H. T., Grimmer H. J., Doss M. K., Hutchinson B. T., Timmermann C., Lyon A., Corlett P. R., Laukkonen R. E. (2024). An integrated theory of false insights and beliefs under psychedelics. Communications Psychology.

[B21-jintelligence-14-00160] Miller G. F., Tal I. R. (2007). Schizotypy versus openness and intelligence as predictors of creativity. Schizophrenia Research.

[B22-jintelligence-14-00160] Morrison R. G., McCarthy S. W., Molony J. M. (2017). The experience of insight follows incubation in the compound remote associates task. The Journal of Creative Behavior.

[B23-jintelligence-14-00160] Moss J., Cranford E. A. (2012). Is insight always the same? A protocol analysis of insight in compound remote associate problems. The Journal of Problem Solving.

[B24-jintelligence-14-00160] Nakagawa S., Schielzeth H. (2013). A general and simple method for obtaining R^2^ from generalized linear mixed-effects models. Methods in Ecology and Evolution.

[B25-jintelligence-14-00160] Nelson T. O. (1984). A comparison of current measures of the accuracy of feeling-of-knowing predictions. Psychological Bulletin.

[B26-jintelligence-14-00160] Prenevost M. H., Reber R., Taku K., Shackelford T. K. (2024). The aha moment: Changes in cognition, affect, motivation, and development. The Routledge international handbook of changes in human perceptions and behaviors.

[B27-jintelligence-14-00160] Salvi C., Bricolo E., Kounios J., Bowden E., Beeman M. (2016). Insight solutions are correct more often than analytic solutions. Thinking & Reasoning.

[B28-jintelligence-14-00160] Smith S. M., Chandolia V. J., Kidd M. A., Paladino M. S. (2026). Generative insight: Aha! moments in category generation and divergent thinking. Journal of Experimental Psychology. General.

[B29-jintelligence-14-00160] Stoffel M. A., Nakagawa S., Schielzeth H. (2021). partR2: Partitioning R^2^ in generalized linear mixed models. PeerJ.

[B30-jintelligence-14-00160] Webb M. E., Laukkonen R. E., Cropper S. J., Little D. R. (2019). Commentary: Moment of (perceived) truth: Exploring accuracy of Aha! experiences. The Journal of Creative Behavior.

[B31-jintelligence-14-00160] Webb M. E., Little D. R., Cropper S. J. (2016). Insight is not in the problem: Investigating insight in problem solving across task types. Frontiers in Psychology.

[B32-jintelligence-14-00160] Webb M. E., Little D. R., Cropper S. J. (2021). Unusual uses and experiences are good for feeling insightful, but not for problem solving: Contributions of schizotypy, divergent thinking, and fluid reasoning, to insight moments. Journal of Cognitive Psychology.

[B33-jintelligence-14-00160] Zedelius C. M., Schooler J. W. (2015). Mind wandering “Ahas” versus mindful reasoning: Alternative routes to creative solutions. Frontiers in Psychology.

