# Supplementary material for: The Aha! Glow: Insight Experiences Bias Judgements of Idea Quality in Divergent Contexts
Source: J Intell. 2026 Aug 1;14(8):160. doi: 10.3390/jintelligence14080160 (PMC13514557; doi:10.3390/jintelligence14080160)
Supplement: Supplementary file 1 [file jintelligence-14-00160-s001.zip › jintelligence-4396172-supplementary.pdf]

Supplementary Material for

The Aha! Glow: Insight Experiences Bias Judgements of Idea Quality in Divergent Contexts

Study 1: Gable et al. (2019) Re-analysis

Below are the remaining results from our re-analysis of the daily diary data from Gable et al. (2019). A linear mixed effect model was run to test the three-way interaction between time (T1 vs. T2), profession (writers vs. physicists) and idea type (Aha! vs. non-Aha) on creativity scores. All three variables were entered as fixed effects, and participants were entered as a random effect (intercepts only).

The main effects of time and idea type replicated analyses from the original study, so we present them here in supplementary materials, along with the main effect of profession and two-way interaction effects.

Main Effects

Idea Type

In accordance with the findings of Gable et al. (2019), the model revealed a significant main effect of idea type, $F(1, 1431.95) = 71.85$, $p < .001$, such that Aha! ideas ($M = 4.52$) were rated as more creative than non-aha ideas ($M = 3.81$). In other words, across all participants and both time points, ideas that arrive with an Aha! feel more creative than ideas that arise through analytic effort - replicating the aha-creativity link established in Gable et al. (2019).

Time

Also in line with the findings of Gable et al. (2019), the model revealed a significant main effect of time, $F(1, 1353) = 11.80$, $p < .001$. Collapsing across profession and idea type, ideas were rated as more creative at T2 ($M = 4.29$) compared to T1 ($M = 4.04$). Overall, creativity ratings increased across the 6-month time period (see Supplementary Table S1).

Profession

The model revealed a significant main effect of profession, $F(1, 93.43) = 10.27$, $p = 0.002$. Collapsing across idea type and time, writers rated their ideas as more creative ($M = 4.48$) than physicists ($M = 3.85$). In the supplementary materials of Gable et al. (2019), writers tended to rate their ideas as more creative than physicists, but at the time of idea generation. Thus, this result extends previously reported findings by showing that writers consistently view their ideas as more creative than physicists do, regardless of the time relative to generation.

Supplementary Table S1

Type III Tests of Fixed Effects from the Profession × Idea Type × Time Linear Mixed-Effects Model on Creativity Ratings

Effect	Numerator *df*	Denominator *df*	*F*	*p*
Profession	1	93.43	10.27	.002
Idea Type	1	1431.95	71.85	< .001
Time	1	1353.00	11.80	< .001
Profession × Idea Type	1	1431.95	0.09	.770
Profession × Time	1	1353.00	16.97	< .001

Idea Type × Time	1	1349.21	12.07	< .001
Profession × Idea Type × Time	1	1349.21	5.53	.019

Note. All effects estimated from a single linear mixed-effects model with random intercepts for participants. Profession was a between-subjects factor; idea type and time were within-subjects factors.

Two-Way Interactions

Idea Type × Time

The model revealed a significant interaction between Idea Type x Time, $F(1, 1349.21) = 12.07$, $p < .001$. At T1, Aha! ideas ($M = 4.52$) were rated 0.96 points higher than non-Aha ideas ($M = 3.56$). At T2, this gap shrank to 0.45 points (Aha! $M = 4.52$; non-Aha $M = 4.07$). This finding was also reported in Gable et al. (2019), and suggests that while Aha! Ideas are rated as highly creative at the moment, this *Aha! glow* fades over time. On the other hand, people come to see their analytic ideas as more creative in hindsight, narrowing the gap between ratings at 6-months follow-up.

Profession × Idea Type

The model revealed that the interaction between Profession and Idea Type was non-significant, $F(1, 1431.95) = 0.086$, $p = .770$. Collapsing across time, writers and physicists show no difference in creativity ratings for Aha! ideas or non-Aha ideas. This finding is consistent with the supplementary materials of Gable et al. (2019), in which they reported there was no difference in the creativity ratings for Aha! ideas versus non-Aha ideas between professions in Study 1.

Profession × Time

The model revealed a significant Profession x Time interaction, $F(1, 1353) = 16.97$, $p < .001$. Writers showed a slight decrease in creativity ratings from T1 to T2 (*M* change = -0.051), regardless of how the idea arrived. On the other hand, physicists show an overall increase in their creativity ratings across time points (*M* change = +0.56). This finding is also in line with the supplementary analysis of Gable et al. (2019); writers tend to downgrade the value of their ideas over time, while physicists upgrade theirs. However, further analysis will reveal that this overall pattern is largely driven by the type of idea.

Study 2: Gender Differences in Insight Phenemonology

We also conducted an exploratory analysis on the Study 2 data to examine gender differences in insight phenomenology on both the AUT and CRAT. The Study 2 sample consisted of 100 total participants (60 female, 39 male, 1 non-binary). As only one participant identified as non-binary in the present research, we restricted analyses to compare insight intensity between participants who identified as male or female. We fit two linear mixed effect models with gender (male vs female) as the predictor, and either AUT insight intensity or CRAT insight intensity as the outcome variable. Participant ID was included as a random intercept in both models.

Notably, the models revealed that there was no significant difference in insight phenomenology between males and females on the AUT, $b = -0.02$, $SE = 0.43$, $t(1481) = -0.05$, 95% CI [-0.87, 0.83], $p = 0.96$; or the CRAT, $b = -0.14$, $SE = 0.39$, $t(2966) = -0.35$, 95% CI [-0.90, 0.63], $p = 0.73$. This suggests that there were no gender differences in the strength of insights experienced during divergent or convergent problem-solving in the present research.
